# HIV Prevalence, Risks for HIV Infection, and Human Rights among Men Who Have Sex with Men (MSM) in Malawi, Namibia, and Botswana

**DOI:** 10.1371/journal.pone.0004997

**Published:** 2009-03-26

**Authors:** Stefan Baral, Gift Trapence, Felistus Motimedi, Eric Umar, Scholastika Iipinge, Friedel Dausab, Chris Beyrer

**Affiliations:** 1 Center for Public Health and Human Rights, Department of Epidemiology, Johns Hopkins School of Public Health, Baltimore, Maryland, United States of America; 2 Center for the Development of People, Blantyre, Malawi; 3 Botswana Network on Ethics, Law, and HIV/AIDS, Gaborone, Botswana; 4 Department of Community Health, University of Malawi,-College of Medicine, Blantyre, Malawi; 5 HIV/AIDS Coordinator, University of Namibia, Windhoek, Namibia; 6 The Rainbow Project, Windhoek, Namibia; 7 Department of Public Health Sciences, University of Toronto, Toronto, Ontario, Canada; Singapore Immunology Network, Singapore

## Abstract

**Background:**

In the generalized epidemics of HIV in southern Sub-Saharan Africa, men who have sex with men have been largely excluded from HIV surveillance and research. Epidemiologic data for MSM in southern Africa are among the sparsest globally, and HIV risk among these men has yet to be characterized in the majority of countries.

**Methodology:**

A cross-sectional anonymous probe of 537 men recruited with non-probability sampling among men who reported ever having had sex with another man in Malawi, Namibia, and Botswana using a structured survey instrument and HIV screening with the OraQuick© rapid test kit.

**Principal Findings:**

The HIV prevalence among those between the ages of 18 and 23 was 8.3% (20/241); 20.0% (42/210) among those 24–29; and 35.7% (30/84) among those older than 30 for an overall prevalence of 17.4% (95% CI 14.4–20.8). In multivariate logistic regressions, being older than 25 (aOR 4.0, 95% CI 2.0–8.0), and not always wearing condoms during sex (aOR 2.6, 95% CI 1.3–4.9) were significantly associated with being HIV-positive. Sexual concurrency was common with 16.6% having ongoing concurrent stable relationships with a man and a woman and 53.7% had both male and female sexual partners in proceeding 6 months. Unprotected anal intercourse was common and the use of petroleum-based lubricants was also common when using condoms. Human rights abuses, including blackmail and denial of housing and health care was prevalent with 42.1% (222/527) reporting at least one abuse.

**Conclusions:**

MSM are a high-risk group for HIV infection and human rights abuses in Malawi, Namibia, and Botswana. Concurrency of sexual partnerships with partners of both genders may play important roles in HIV spread in these populations. Further epidemiologic and evaluative research is needed to assess the contribution of MSM to southern Africa's HIV epidemics and how best to mitigate this. These countries should initiate and adequately fund evidence-based and targeted HIV prevention programs for MSM.

## Introduction

While southern Sub-Saharan Africa has long been the most HIV/AIDS affected region globally, it has been arguably the most understudied for the risk of HIV associated with male to male sexual contact [1]–[3] The crude characterization of these epidemics as generalized and driven by heterosexual risks has obscured the component of Southern Africa's epidemics which may be due to risks among men who have sex with men (MSM). The marked homophobia, discrimination, and criminalization of same-sex behavior in much of Africa have likely limited investigation among these men. [4], [5]. Data regarding the prevalence of MSM in the region are among the sparsest globally, but there is evidence that male to male sexual contact is a reality on this continent as on all others [2]. To date, there have been published papers from only Senegal and Kenya describing HIV risk and prevalence among MSM in Africa [6], [7]. However, a systematic review found studies from other African countries either not presenting HIV prevalence data or studies that to-date have only been presented as abstracts [3]. These studies suggest that African MSM are at substantial risk for HIV infection, and that they have been markedly underserved and marginalized. Reported HIV rates, where available, have been higher than among other men of reproductive age in the same populations, yet these men tend to have limited knowledge of the health related risks of anal intercourse [8]–[10]. The lack of data on MSM and HIV are paradoxically the most marked for the world's highest prevalence zone; the southern region of Sub-Saharan Africa. No published studies have reported HIV prevalence among MSM in Namibia, Malawi, and Botswana, three profoundly HIV/AIDS affected southern states. MSM have not been included in the HIV/AIDS strategies in these countries and same sex behavior among consenting adults is criminalized in all three states in 2008.

Concurrency of sexual relationships has been posited by several groups as a key driver of the high rates of prevalence in the southern African region [11], [12]. Yet concurrency of same and opposite sex partners has been little studied, and may play important roles as well.

To address these lack of HIV prevalence and risk and rights data among MSM in these states, and to support the emerging community groups advocating for recognition and health services for these men, our collaborative group developed a technically simple epidemiology and human rights study protocol which could be implemented by LGBT (Lesbian, Gay, Bisexual, and Transgender) rights groups with minimal cost, and with maximum protection for participants. The results presented here are the first epidemiologic probe of HIV among MSM in Namibia, Botswana, and Malawi.

## Methods

### Study Area

This study was completed in Blantyre and Lilongwe in Malawi, Windhoek, Namibia, and Gaborone, Botswana. These countries were chosen based on being within the encashment area of the Open Society Initiative for Southern Africa, having generalized HIV epidemics, no data available characterizing HIV risk among MSM, and having community-based organizations that were keen and able to collaborate on a study characterizing MSM in their community.

### Study population and sampling methods

Eligible participants were 18 years old or older, had a history of ever having had anal intercourse with another man, and were able to give verbal informed consent for HIV screening in local languages. Inclusion criteria were not based on sexual orientation or identity, frequency of sexual contacts, previous HIV testing, or known HIV serostatus. Given the hidden nature of MSM in these communities, participants were recruited by in-country community-based organizations (CBO) with experience working with gay, bisexual, and other MSM. In-country technical support was provided as requested by the CBOs. In Namibia, investigators from the University of Namibia HIV/AIDS unit played a central role in providing ongoing support for this work. Similarly, researchers from the Malawi College of Medicine supported the Malawian CBO. The study staff was provided on–site training in outreach and recruitment, obtaining informed consent, and in interviewing techniques. The study was anonymous, confidential, and no written communications were shared with participants to minimize the risk of disclosure of MSM status. Sample size calculations were based measuring risk associated with unprotected anal intercourse (UAI). Assuming that UAI increases risk of HIV transmission by approximately 80% with a significance level of 0.05 and a power of 80%, the minimum necessary sample size was 150 men[13]. Rounding up, the planned sample size was 200 for each of the three sites for a total of 600 men.

Given the lack of gay venues, recruitment was done through snowball sampling. In Malawi, 20 seeds were identified by the local CBO, Center for Development of People (CEDEP), and each of the seeds recruited either 9 or 10 participants resulting in a total sample size of 202. In Namibia, 20 seeds were identified by the local CBO, the Rainbow Project (TRP) and through chain-referral recruited 20 participants each for a total sample size of 218. In Botswana, the partner was the Botswana Network on Ethics, Law, and HIV/AIDS (BONELA), who recruited 10 seeds. However, ultimately only 117 MSM were accrued in Botswana because of difficulty in accessing this population and significant delays in the local approval processes.

### HIV Screening

Saliva samples were obtained for anonymous rapid HIV screening among interviewees. Oral fluid HIV was done testing using the OraSure Oraquick HIV-1/2 kit (Orasure Technologies, Bethlehem, PA, USA), licensed by the US FDA, with a sensitivity of 99.1% for oral fluid (compared to 99.7% with serum), and a specificity of 99.6% with oral fluid (compared to 99.9% with serum) [14]. This HIV screen was for study purposes, not for confirmative diagnosis of HIV infection: participants were encouraged to seek appropriate venues for HIV counseling and testing.

### Study Instrument and Interviews

A short structured survey instrument containing 45 questions was developed with a modified Delphi Method including experts in determinants of health, HIV epidemiology, and human rights. The instrument was piloted with MSM CBO members in each of the countries, and revised and locally adapted. Interviews took approximately 25 minutes to complete, and collected no identifiable information. After the interview, the oral fluid sample was obtained and the study participants were remunerated at different levels (between 5–10 USD) as determined by the partner CBO for their time and transportation costs. To maintain confidentiality and anonymity of the participants in the study, two separate rooms were required to ensure that the person reading the test result did not make direct contact with the respondent. Instead, non-traceable alphanumeric participant codes linked the HIV screening data to the surveys.

### Statistical Analysis

Survey instruments were linked anonymously to HIV testing results using participant codes. Data were doubly entered into Microsoft Excel and subsequently imported to Stata 9.2 for analysis[15]. Univariate analyses included two-sample tests for differences in proportions, χ^2^ tests of independence, and logistic regression assessing the relationship between risk factors and HIV status. Backward elimination with a p-value set to 0.1 was used to determine which variables were included in the multivariate model. .In the multivariate logistic regression models, variables that were significantly (p<0.05) or moderately significantly (p<0.1) associated with HIV status were reported by presenting adjusted odds ratios (aOR) with 95% confidence intervals.

The study was approved by the Institutional Review Board of the Johns Hopkins Bloomberg School of Public Health and the University of Namibia, and the Ministry of Health in Botswana. Ethics approval was also sought from the National AIDS Council (NAC) in Malawi. While receipt of the application was confirmed on numerous occasions over many months, no answer was given. A thorough consultation with the MSM community in Malawi demonstrated overwhelming support to move forward with the study. And since the protocol was identical to that approved by the two other in-country human subjects committees, CEDEP employed an internal review mechanism and approved the study.

## Results

### Sociodemographics and Sexual Practices of study participants

The participants tended to be young overall with mean ages of 24–26 in each of the three countries (Table 1). The majority had at least a secondary education, and approximately half were currently employed. There were high levels of bisexual concurrency observed, defined as concurrent regular partnerships with both men and women, but was most common in Malawi (p<0.05).

**Table 1:** Selected characteristics of MSM in Malawi, Namibia, and Botswana.

Characteristic		Malawi	Namibia	Botswana	Pooled
Age	Mean/Median (Range)	25.6/ 25 (Range 19–49)	24.4/23 (Range 18–52)	25.8/24 (Range 18–49)	24.9/24
Rural Origin		30.4% (61/201)	42.6 (93/218)*	23.1% (27/117)	33.8% (181/536)
Education	Primary or less	0.5% (1/199)	9.6% (21/218)*	1.7% (2/117)	4.5% (24/534)
	Secondary or more	99.5% (198/199)	90.4 (197/218)*	98.3% (115/117)	95.5% (510/534)
Currently Employed	Employed	51% (102/200)	41.9% (91/218)	49% (57/117)	46.7%( 250/535)
Current Relationship	Only Regular Male Partner	28.6% (56/196)	34.6% (74/214)	45.6% (52/114)*	34.7% (182/524)
	Only Regular Female Partner	21.9% (43/196)	17.3% (37/214)	10.5% (12/114)*	17.5% (92/524)
	Concurrent Regular Male and Female Partner	25.5% (50/196)*	10.3% (22/214)	12.3% (14/114)	16.4% (86/524)
Self –Reported Sexual Orientation	Heterosexual	6.5% (13/200)	19.4% (42/216)*	3.4% (4/117)	11.1% (59/533)
	Gay/Homosexual	40.5% (81/200)	48.6% (105/216)	66.7% (78/117)*	49.5% (264/533)
	Bisexual	53% (106/200)*	29.1% (63/216)	29.1% (34/117)	38.1 (203/533)
	Transgender	0 (0/200)	2.88% (6/226)	0.9% (1/117)	1.3% (7/533)
Disclosed sexual orientation to	At least one Family member	17% (34/200)*	44.5% (97/218)	60.3%( 70/116)	37.6% (201/534)
	Any one Health Care Worker	8.96% (18/201)*	21.6% (47/218)	24.1% (28/116)	17.4% (93/535)
	Family or Health Care Worker	20.5% (41/200)*	50.0% (109/218)	64.7% (75/116)	42.1% (225/534)
In last 6 months:	Number of Male Partners (Mean/Median) (Range)	3.9/2 (Range 0–52)	2.9/2 (Range 0–30)	2.8/2 (Range 0–24)	3.2/2 (Range 0–52)
	Number of men with > = 5 partners	17.54% (30/171)	14.7% (31/211)	12.8% (15/117)	15.2% (76/499)
	Number of female partners (Mean/Median) (Range)	1.5/1 (Range 0–12)	1.2/1 (Range 0–12)	0.7/ 0 (Range 0–7)	1.2/1 (Range 0–12)
	Both male and female sexual partners in last 6months	63.44% (118/186)*	50.7% (108/213)	43.6% (51/117)	53.7% (277/516)
	Have injected illegal drugs (IDU)	12.2% (18/147) 48 refused to answer	8% (16/200) 20 refused to answer	3.4% (4/88) 29 refused to answer	8.7% (38/435) 97 refused to answer
	Found male partner on internet	44.2% (88/199)	38.5% (84/218)	56.9% (66/116)*	44.7% (238/533)
Self-reported biggest threat to health	HIV	84.7% (161/190)	58.9% (119/202)*	80.9% (89/110)	73.5% (369/502)
	Sexually Transmitted Infections	5.3% (10/190)	7.4% (15/202)	2.7% (3/110)	3.6% (28/502)
	Malaria/Tuberculosis	3.2% (6/190)	4.5% (9/202)	0.9% (1/110)	3.2% (16/502)
	Violence	2.6% (5/190)	15.4% (31/202)*	3.6% (4/110)	8.0% (40/502)

* Statistically significant difference of proportions (two-sided) for each variable between the three countries (p<0.05).

In all three countries, MSM had more male sexual partners than female sexual partners with a mean of between 1–1.5 female sexual partners in last 6 months, again positively skewed. MSM reported medians of between 3–4 male sexual partners over the preceding 6 months, though the distributions were positively skewed for all three countries with a minority of men reporting large number of male partners. Active bisexual practices–both male and female sexual partners in the same time frame was common across all three countries, but again, was most common in Malawi (p<0.05).

Disclosure of sexual orientation to family was more common in both Namibia and Botswana than Malawi (p<0.05). In Malawi, less than 10% of respondents disclosed their sexual orientation at any interaction with a health care worker, and the rate was below 25% in both Namibia and Botswana.

8.7% (38/435) of MSM admitted to injecting drugs, but more participants refused to answer this question than any other (18.1% - 97/536). The total sample size varied because participants refused to answer certain questions of the survey instrument.

In the pooled analysis, 44.7% (238/533) had used the internet to find a male sexual partner in the last 6 months, with the highest rates being in Botswana (p<0.05). Across all three sites, the biggest self-reported risk to one's health was from HIV/AIDS, though 8.0% of the participants considered violence as the most important threat to their personal health. Compared to Malawi and Botswana, MSM in Namibia were less likely to consider HIV/AIDS as the biggest threat to their health (p<0.05), and most likely to consider violence as the single biggest threat to their health (p<0.05).

### HIV Related Knowledge

Men were more likely to have received any information about how to prevent HIV infection from women than from men in all three sites (p<0.05) (Table 2). Men were more likely to know that HIV can be transmitted by vaginal intercourse than by anal intercourse in both Botswana and Malawi (p<0.05). In the pooled analysis, 85.3% (44/516) knew that HIV could be transmitted through injecting drug use. Only 70.3% of men in Malawi knew that HIV could be transmitted by these three modalities, whereas this was again higher in Botswana and Namibia (p<0.05), predominantly because of the dearth of knowledge about IDU.

**Table 2:** Levels of HIV-related knowledge among MSM in Malawi, Namibia, and Botswana.

Characteristic		Malawi	Namibia	Botswana	Pooled
Received any information about preventing HIV infection from	Women	94.5% (189/200)	95.0%(207/218)	90.6% (106/117)	93.8% (503/536)
	Men	56.5% (113/200)*	84.9%(185/218)*	50.4% (59/117)*	66.7% (357/535)*
Know that HIV can be transmitted via	Anal Intercourse	92.3% (180/195)	94.3% (200/212)	93.1% (108/116)	93.1% (488/524)
	Vaginal Intercourse	98.5% (197/200)	96.3% (208/216)	99.2% (116/117)	97.8% (521/533)
	Injecting Drug Use	74.35% (142/191) *	91.5% (194/212)	92.0% (104/113)*	85.3% (440/516)*
	All three above	70.27% (130/185)	87.8% (180/205)	87.5% (98/112)	81.1% (408/536)

*Statistically significant difference of proportions (two-sided) within each country and in pooled analysis (p<0.05).

### HIV Risk Factors

Always using condoms among MSM with male or female partners was equivocal in Malawi and Botswana, but in Namibia, MSM were more likely to use condoms with men than women (p<0.01). In Namibia and Botswana, MSM were more likely to always use condoms with casual partners as compared to their regular sexual partners (p<0.05), whereas condom use between casual and regular partners was equivalent in Malawi. Of those who used lubricants during anal intercourse, a minority (38.2%, 130/340) overall used water-based lubricants as compared to petroleum-based products including petroleum jelly, fatty and body creams, with highest rates of WBL use in Botswana (50.7%, 36/71, p<0.05). Finally, only 3.3% (13/389) of the study sample were practicing safe anal sex as defined by always using condoms and water-based lubricants.

Transactional sex, as defined by anal intercourse in exchange for money or gifts with a casual partner, was common across all three sites. Overall, it was more common in Malawi (62.6% - 124/198), then Namibia (37.3%-81/217), and least prevalent in Botswana (29.3% - 34/116) (p<0.05). In Malawi, the sample was more likely to have received money/gifts for anal intercourse, but this difference was not found in the sample in Namibia or Botswana. MSM had been most commonly previously tested for HIV in Botswana (82.9% 97/117), followed by Namibia (59.4%- 129/217), and then Malawi (35.2% 69/196) (p<0.05). 18.5% (40/216) of MSM had ever been told by a health care worker that they had a STI in Namibia, whereas 8.5% (17/199) of MSM in Malawi had received this diagnosis similar to 9.4% (11/117) of MSM in Botswana.

### Human Rights Contexts

Human rights abuses among MSM in the study sample were prevalent across all three countries. Between 5–10%, depending on the site, of the study participants had been denied housing in the past for reasons other than the ability to pay (Table 3). Being afraid to seek health services because of sexual orientation was reported by 17.6% (35/199) in Malawi, 18.3% (40/218) in Namibia, and 20.5% (24/117) in Botswana. While having been denied health care was less common with a pooled prevalence of 5.1% (27/533), disclosing sexual orientation to a health care worker was significantly associated with having been denied health care (OR 4.2,95% CI 1.9–9.3).

**Table 3:** The prevalence of human rights abuses among MSM in Malawi, Namibia, and Botswana.

Characteristic	Malawi	Namibia	Botswana	Pooled
Denied housing other than not being able to pay	6.5% (13/200)	8.3% (18/218)	5.2% (6/116)	6.9% (37/534)
Denied health care based on sexuality	4.02% (8/199)	8.3% (18/217)	0.85% (1/117)	5.1% (27/533)
Afraid to seek health services	17.59% (35/199)	18.3% (40/218)	20.5% (24/117)	18.5% (99/535)
Afraid to walk in community	15.5% (31/200)	16.7% (36/215)	29.1% (34/117)	19.0% (101/532)
Blackmailed because of sexuality	18.00% (36/200)	21.3% (46/216)	26.5% (31/117)	21.2% (113/533)
**Yes to any of the above related to sexuality**	34.34% (68/198)	41.5% (88/212)	56.9% (66/116)	42.1% (222/527)
Beat up by government or police official	8.08% (16/198)	21.7% (47/217)	1.7% (2/117)	12.2% (65/533)

MSM reported being afraid to walk down streets in their own community most commonly in Botswana , but also to a lesser extent in Malawi and in Namibia (p<0.05). Overall 42.1% (222/527) of MSM answered yes to any of these markers of human rights violation. 12.2% (65/533) of the total sample indicated that they had been physically abused by a government or police official, with the highest rates in Namibia (p<0.05). Finally, 11.4% (61/534) of the sample reported ever having been raped by another man, with similar rates across the three sites.

Blackmail or extortion on the basis of sexual orientation or behavior was quite prevalent in the sample with an overall rate of 21.2%. In the pooled analysis, univariate associations with blackmail included having either paid or received money or gifts for casual sex (p<0.01); having told a member of the family of one's sexual orientation (p<0.01); and having a told a clinic or health care worker of one's sexual orientation (p<0.05), and not having had an HIV test in the preceding 6 months (p = 0.06) (data not shown). Multivariate analysis was completed adjusting for these covariates and blackmail was significantly associated with having taken part in transactional sex (aOR 2.5,95%CI 1.6–3.8), not having had a HIV test in last 6 months (aOR 0.56,95%CI 0.3–1.0), having disclosed same sex behavior to a member of the immediate or extended family (aOR 2.3,95% CI 1.4–3.6), but not to health care workers (aOR 0.9,95%CI 0.5–1.6).

### Associations with HIV Infection

The overall HIV prevalence was 17.4% (93/536); however, there was significant variation of HIV prevalence with increasing age (Table 4). The HIV prevalence among those between the age of 18 and 23 was 8.3% (20/241), then 20.0% (42/210) among those 24–29, and 35.7% (30/84) among those older than 30. Overall, 23.7% (22/93) were aware of their HIV status, though this varied significantly between countries (p<0.05). In Malawi, more than 95% were unaware of their status, whereas in Botswana 76.3% were unaware and in Namibia, 41.8% were unaware of their status.

**Table 4:** The prevalence of HIV among MSM in Malawi, Namibia, and Botswana and proportion aware of serostatus.

HIV Prevalence	Malawi	Namibia	Botswana	Combined
	Estimate (n – 95% CI)	Estimate (n - 95% CI)	Estimate (n - 95% CI)	Estimate (n - 95% CI)
All Ages	21.4% (43/201 - 16.3–27.6)	12.4% (27/218 – 8.7–17.4 )	19.66% (23/117 – 13.5–7.8)	17.4% (93/536 – 4.4–20.8 )
Age 18–23	15.2% (12/79 – 8.9–24.7)	3.5% (4/113 – 1.4–8.8)	8.2% (4/49 – 3.2–19.2)	8.3% (20/241 – 5.4–12.5)
Age 24–29	21.6% (19/88 – 14.3–31.3)	17.1% (12/70 – 10.1–27.6)	21.2% (11/52 – 12.2–34.0)	20.0% (42/210 – 15.2–25.9)
Age > = 30	35.3% (12/34 – 21.5–52.1)	31.4% (11/35 – 18.6–48.0)	46.7% (7/15 – 24.8–70.0 )	35.7% (30/84 – 26.3–46.4)
Aware of HIV status	4.7% (2/43)	59.2% (16/27)	17.4% (4/23)	23.7% (22/93)

Univariate predictors varied between countries and can been seen in Table 5. In the pooled analysis, increasing age, being employed, not always wearing condoms with men, casual and regular partner, having been diagnosed with an STI, and having had transactional sex were significantly associated with HIV (p<0.05). Furthermore, self-reporting as homosexual or bisexual compared to heterosexual was associated with HIV (p = 0.06). In the multivariate model, ever having been diagnosed with an STI, being older than 25 (aOR 4.0, 95% CI 2.0–8.0) and not always wearing condoms (aOR 2.6, 95% CI 1.3–4.9) were significantly associated with being infected with HIV in the pooled analysis (Table 6). Country-specific associations also included having been diagnosed with an STI was strongly linked to being HIV-positive (aOR 33.7, 95% CI 3.4–148.2) in Botswana and having used the internet to find male sexual partners in Malawi (aOR 3.6 95% CI1.0–13.7).

**Table 5:** Univariate associations with HIV status among MSM in Malawi, Namibia, and Botswana and proportion aware of serostatus.

Characteristic	Malawi	Namibia	Botswana	Combined
	OR (95% CI)	OR (95% CI)	OR (95% CI)	OR (95% CI)
Increasing Age Groups	**1.7 (1.1–2.8)**	**7.3 (2.6–20.1)**	**3.2 (1.2–8.2)**	**2.6 (1.6–4.2)**
Self-reported homosexual orientation	All self-reported homosexual MSM were HIV positive	1.4 (0.4–4.2)	0.7 (0.1–7.4)	2.4 (0.9–6.3)
Being employed	0.9 (0.5–1.8)	0.8 (0.4–1.9)	**5.0 (1.7–14.5)**	**1.7 (1.1–2.6)**
Not always wearing condoms with men	**6.4 (1.4–28.5)**	**2.8 (1.1–6.8)**	0.6 (0.2–1.7)	**2.2 (1.3–3.8)**
Not always wearing condoms with women	1.3 (0.3–4.7)	3.7 (0.7–18.6)	1.8 (0.3–11.0)	2.3 (0.9–5.4)
Not always wearing condoms with casual partners	7.0 (0.9–53.7)	**17.7 (2.4–133.3)**	0.6 (0.2–2.3)	**4.9 (2.1–11.4)**
Not always wearing condoms with regular partners	5.6 (0.7–43.4)	**8.3 (1.1–62.9)**	2.0 (0.6–7.6)	**4.3 (1.7–10.9)**
Used internet to find male partner	1.8 (0.9–3.6)	0.9 (0.4–2.1)	0.9 (0.4–2.3)	1.3 (0.8–2.0)
Having been ever diagnosed with a STI	1.6 (0.5–4.8)	**2.9 (1.2–7.2)**	**16.0 (3.8–67.2)**	**2.7 (1.5–4.8)**
Had transactional sex	1.5 (0.7–3.1)	1.2 (0.5–2.7)	**2.8 (1.1–7.2)**	**1.7 (1.1–2.7)**
Ever arrested	**2.4 (1.0–5.9)**	0.8 (0.3–1.8)	All MSM who reported arrest were HIV positive	0.8 (0.5–1.4)
Having been raped	3.3 (0.8–14.7)	1.5 (0.5–4.3)	**6.3 (1.5–25.6)**	1.1 (0.6–2.2)

• Bolded are statistically significant (p<0.05).

**Table 6:** Multivariate adjusted associations with HIV status among MSM in Malawi, Namibia, and Botswana and proportion aware of serostatus.

Characteristic	Malawi	Namibia	Botswana	Combined
	aOR (95% CI)	aOR (95% CI)	aOR (95% CI)	aOR (95% CI)
Older than 25	**3.6 (1.1–11.1)**	**4.9 (1.5–16.2)**	**7.2 (1.2–41.9)**	**4.0 (2.0–8.0)**
Not always wearing condoms with men	**17.1 (1.9–149.8)**	6.7(0.8–54.1)	0.92 (0.3–2.9)	**2.6 (1.3–4.9)**
Ever Diagnosed with an STI	2.33 (0.2–23.1)	0.55 (0.2–1.9)	**33.7 (3.4–148.2)**	0.72 (0.3–1.6)
Used Internet to find male partners	**3.6 (1.0–13.7)**	1.52 (0.5–4.9)	1.1 (0.4–3.3)	0.68 (0.4–1.3)

• Bolded are statistically significant (p<0.05).

## Discussion

This is the first study to investigate HIV status and risks for HIV infection among MSM in Namibia, Botswana, and Malawi. It is also the first attempt, to our knowledge, to evaluate the human rights contexts among MSM and to link individual level rights abrogation to HIV biological outcomes in the African context.

Overall, HIV rates were substantial, and risks for HIV infection from sex with both were men and women were common. The participants were generally young, though there was a significant association between HIV and age. Excluding the few men above the age of 49, overall more than one-third (35.7%, 95%CI 26.3–46.4) of MSM between the ages of 30–49 were HIV infected. These data suggest that this is not a new epidemic of HIV among African MSM which is spreading more rapidly among younger MSM, as has been seen observed among MSM in other settings such as Russia [16]. Because younger men were much less likely to be HIV infected, prevention programs targeting younger MSM in these populations could have marked potential for avoiding future infections. All possible combinations of biomedical and behavioural interventions need to be evaluated including those directed at MSM who are already HIV seropositive[17]. While very little is known about the benefit of targeted HIV prevention programming among MSM in Africa, in other contexts these approaches are known to be very effective in decreasing unprotected anal intercourse (UAI) [18], [19]. Prevention research and optimization of existing prevention tools for MSM are a clear public health priority for Southern Africa.

Approximately two-thirds of MSM had received any information about preventing HIV infection from other men, which was higher than expected. However, given that these men were largely recruited from within the same networks of men who are served by these CBOs, this likely overestimates the men exposed to this information in each country. Basic knowledge and condom access and availability are necessary for increased condom usage, but not sufficient. Recent studies have demonstrated that African MSM are less likely to have UAI if they use water-based lubricants (WBL), have been counseled about the risks of UAI, and more likely to have UAI if they regularly drink alcohol or do not know that HIV can be transmitted via anal intercourse [8], [10]. Understanding condom use among MSM in the African context is especially relevant as in all three countries, not always wearing condoms was highly predictive of being HIV positive. If safe sex is defined as the usage of WBL in addition to always wearing condoms, then less than 1 in 20 MSM practiced safe sex in this study. The more common use of oil-based products, including vaseline and body/fatty creams appears partly due to cost and partly to availability. Increasing the availability of affordable and practical WBL should be a key focus of prevention strategies.

A significant proportion of MSM self-identified as either heterosexual or bisexual, and many were married or had at least one female sexual partner in the preceding six months. These results were consistent with a previous knowledge, attitudes, and perceptions study of MSM in Malawi[20]. Concurrency of sexual relationships, which has been posited by many investigators as a key driver of heterosexual transmission in this region, appears to be relevant to MSM as well[11], [12]. Some 17% of men overall were in concurrent stable relationships with men and women and over half of the respondents had both male and female sexual partners in previous 6 months, suggesting that concurrency of sexual relationships which include both same and opposite sex partnerships may be an under—appreciated component of HIV spread in this region.

Approximately one tenth of men reported the injection of illegal drugs. There is an increasing appreciation that IDU behavior is also a reality in the African context, and more work is needed to better characterize this risk and its relationship to sexual risk exposures among African men [21].

The use of the internet to find male sexual partners was common across all three countries with nearly half of the respondents reporting using the internet for this purpose. In settings where homosexuality is criminalized and the police harass MSM, with no open venues for gay people to congregate, the internet has preceded the development of openly gay physical venues. Given the hidden nature of this population, the internet may represent a powerful tool in efficiently accessing and delivering HIV prevention education to these men [22].

Self-reported sexual orientation as homosexual or bisexual compared to heterosexual was significantly associated with HIV. While not explored here, this differential risk between identities may relate to sexual positioning, and will be relevant to HIV prevention programming [23]. Disclosure of sexual orientation to either any one member of their immediate or extended family, or any one health care worker was very low. These are hidden populations of men, currently only accessible for study and prevention programming through sexual and social networks with other MSM. In Kenya, where being MSM has become more of an accepted identity, the MSM community continues to evolve a gay identity and become more socially visible [24]. While there is a real risk for backlash, the self-identification of these men and community development may allow for better dissemination of education and prevention measures.

This study served as an assessment of human rights contexts for MSM in these countries. The results are a powerful reminder of the level of stigma, discrimination and human rights abuses that these men face in their everyday lives, including being denied housing and healthcare, being afraid to walk down the streets of one's community, or being afraid to seek health care services. Though each of these rights abrogation likely limit access to HIV preventive services, none were significantly associated with HIV at the individual level. This could have been because abrogations were so common that ceiling effects made attribution difficult, as well as the fact that country sample sizes were small. However, having disclosed sexual orientation to family members was significantly associated with blackmail, and, having disclosed sexual orientation to a health care provider was significantly associated with having been denied health care. In the short term, these two factors will continue to limit disclosure of sexual orientation. In addition, those who reported blackmail were also less likely to have been tested for HIV in last 6 months. These structural barriers to available health care services will limit the efficacy of any interventions targeting individual level determinants of HIV transmission among MSM and must arguably, be mitigated to effectively decrease HIV incidence [25].

There are several limitations to this cross-sectional study. Resources and the constraints of working with small CBOs in these rights constrained environments limited the scale and scope of these probe studies. Due to the nature of the study we were unable to establish directions of causality. There are known biases in questionnaire-based estimates of sexual violence [26]. Specifically, using narrowly defined terms of sexual violence such as rape in a study instrument, as was done in this study, may underestimate its prevalence. The study samples are convenience samples generated by use of chain-referral techniques rather than population-based samples, which is a key limitation with this study methodology and limit the generalizability of the results to the wider population of MSM in respective countries. This problem, referred to as homophily, will likely be best addressed by larger respondent-driven sampling (RDS) studies, and by venue-time sampling approaches, where feasible[27]. Even with RDS or venue-based sampling, there will be biases in the sample recruited and calculated estimates, though likely of lesser magnitude than when using convenience samples. Non-random sampling may also have overestimated the level of HIV-related knowledge seen in the results. Finally, MSM tend to congregate in urban areas, which is why recruitment took place in urban centers; again, this may limit generalizability.

One conclusion of this research perhaps bears stating openly: MSM exist in Malawi, Namibia, and Botswana, and are at high risk for HIV infection and human rights abuses. Piot et al. recently published a call to action for HIV prevention indicating that each country should appropriate HIV prevention expenditures in an evidence-based manner [28]. To date, there have been no dedicated government expenditures funding evidence-based and targeted HIV prevention programs for MSM in these three countries. To comprehensively address the HIV epidemic, African national AIDS strategies should allocate funds based on evidence such as presented here, ensuring that the right to health care is respected for all. Community partners willing and able to do this challenging work also exist, and supporting these partners and including them in HIV/AIDS fora in country and internationally is likely critical to the success of prevention, treatment, and care programs in these countries.
